# Scoring the correlation of genes by their shared properties using OScal, an improved overlap quantification model

**DOI:** 10.1038/srep10583

**Published:** 2015-05-27

**Authors:** Hui Liu, Wei Liu, Ying Lin, Teng Liu, Zhaowu Ma, Mo Li, Hong-Mei Zhang, Qing Kenneth Wang, An-Yuan Guo

**Affiliations:** 1 Department of Biomedical Engineering, Key Laboratory of Molecular Biophysics of the Ministry of Education, College of Life Science and Technology, Huazhong University of Science and Technology, Wuhan, Hubei, 430074, PR China; 2 Key Laboratory of Molecular Biophysics of the Ministry of Education, College of Life Science and Technology and Center for Human Genome Research, Huazhong University of Science and Technology, Wuhan, 430074, China; 3 Center for Cardiovascular Genetics, Cleveland Clinic, Cleveland, OH, USA

## Abstract

Scoring the correlation between two genes by their shared properties is a common and basic work in biological study. A prospective way to score this correlation is to quantify the overlap between the two sets of homogeneous properties of the two genes. However the proper model has not been decided, here we focused on studying the quantification of overlap and proposed a more effective model after theoretically compared 7 existing models. We defined three characteristic parameters (*d, R, r*) of an overlap, which highlight essential differences among the 7 models and grouped them into two classes. Then the pros and cons of the two groups of model were fully examined by their solution space in the (*d, R, r*) coordinate system. Finally we proposed a new model called OScal (Overlap Score calculator), which was modified on Poisson distribution (one of 7 models) to avoid its disadvantages. Tested in assessing gene relation using different data, OScal performs better than existing models. In addition, OScal is a basic mathematic model, with very low computation cost and few restrictive conditions, so it can be used in a wide-range of research areas to measure the overlap or similarity of two entities.

Constructing kinds of gene networks is a common and important task in biological study, which focuses on assessing the relationships of all gene pairs. The networks serve as important approaches to a hotspot research, elucidating molecular mechanisms underlying complex phenotypes^1^,^2^,^3^, such as complex diseases and traits. Since they are influenced by interaction of multiple genetic and environmental factors^4^,^5^,^6^,^7^, thus to study them two major questions need to be addressed: what are causative/risk factors and what kind of functional relationship lies between them^8^,^9^. Studies have shown that a gene network is helpful to such two problems via its capacity in predicting disease genes^10^,^11^ and discovering the functional module among genes. A gene network is a representative form of all gene relations, and the relation of two genes would be physical interaction, co-expression, or functional association assessed by integrating multiple kinds of curated data^12^. Functional association of genes could be assessed by many computational approaches, which are based on a common idea that genes associated with the same or related disease phenotypes tend to participate in a common functional module (such as protein complex or pathway)^13^,^14^,^15^.

Three typical kinds of methods are based on semantic similarity^16^,^17^,^18^,^19^, set theory^20^, Bayesian^21^ or SVM^22^ classification respectively. Among them using set theory is an intuitive and prospective one^20^, which generates a score vector for every pair of genes based on integration of multiple kinds of data. Fig. 1 shows the general work flow of such scoring system, and it has many advantages. First, it is free of a pre-selected golden standard on which SVM or Bayesian method are based, since up to now there is no high-quality set of established functional associated gene pairs. Second, it is very basic and could be used to integrate many heterogeneous kinds of data. Third, the scores on different dimensions show the detailed associations of two genes at different aspects. In addition if golden positives are given, the score vector could be transformed into a digital overall score further. However this method depends on a proper model used to measure the overlap, in other words, it is an important and critical task to choose a proper function *f* as shown in Fig. 1. But we still don’t know which function is a proper one, thus this issue needs to be settled first.

There emerged many such functions over the past years since quantifying the overlap of two sets is an old and general question. Overlap coefficient (C) is used to measure the overlap between two sets. The Jaccard index (J)^23^ and the Ochiai coefficient (K)^24^ are popularly used to measure similarity of two sets. The Hypergeometric (H) and Binominal (B) distribution are commonly used to measure the enrichment significance between two gene lists^25^. Poisson distribution (P) is an approximation of model H and B^26^. Mutual information (I) is a measure of the variables’ mutual dependence^27^. As shown in Fig. 1(a) an overlap of two sets can be exclusively described by a triple (*d, m, n*) (we focused on comparing overlaps in the same background in this study, so *N* is constant) and its score is the function value (*f*-value). The letter in the bracket behind every model was used to represent the model and its function symbol, and the expressions of 7 models are Equations (1~8) in Method. Model H, B and P calculate the occurrence probability (p-value) of the overlap, and we used –log (p-value) as score in our study.

Different functions generate different scores for the same overlap. Fig. 1(a) shows some examples of overlaps, and their scores calculated by the 7 models were listed in Table 1. The p-value of case O2 calculated by model H and B exceed the infimum (inf) of an ordinary computer and are taken as 0, and then their scores (–log (p-value)) are Inf. The score calculated by model P in this study is exactly calculated by its simplified form (see Method). All models are consistent with each other when comparing obviously different cases (case O2 vs. O6 in Fig. 1(a)), but sometimes they are contradictory (case O3 vs. O5). Then what are the differences among these functions (models)? Which one should be better in assessing functional association of genes? Rare studies focus on these questions except for those compared the performances of some similarity coefficient (including model J and K and so on) in certain specific application^28^.

Here we compared their expressions and solution spaces, aiming to theoretically investigate their difference. We discovered three characteristic parameters (*d, R, r*) of an overlap, which highlight some essential differences among these models, i.e. their different weights on *d*, *R* and *r*. Such difference separates them into two classes: model J, K and C are models in class I, model H, B, P and I are models in class II. Then we compared the performances of these two classes in assessing gene relationship, and hypothesized that gene pairs with high correlation scores have high probability to be functional related gene pairs. In other words, a good scoring model should generate scores that have good correlation with the precision. The pros and cons of the two groups of model were fully examined by their solution space in the (*d, R, r*) coordinate system. Based on these findings, we proposed a new model called OScal (Overlap Score calculator), which is designed to achieve a good balance between these two classes.

## Result

### The characteristic parameters highlight the essential difference among models

**Figure 1:**
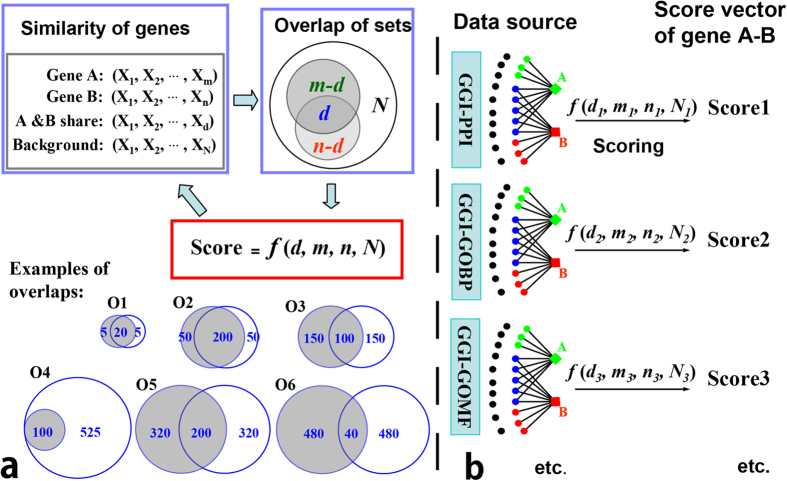
Philosophy and workflow for scoring gene correlation by overlap. (**a**) Using one kinds of data (such as PPI) to score gene relation, every gene could be represented by a set of its properties (PPI partners). Then the association of two genes at this aspect could be denoted by the overlap of the two corresponding sets. One set has *m* elements and the other has *n* elements, and they share *d* elements. The overlap could be quantified by a certain function *f*, and its score is the function value, which is also the score of the gene relation. Different overlaps have different scores, showing different relation among different gene pairs. Using one data source, the background number is known and constant, and it is not a variable. (**b**) Using one data source, two genes could obtain a score at that aspect; when integrated multiple data sources, the relation of two genes could be denoted as a multi-dimension vector. Using different data source, the background number will be the fourth variable.

**Figure 2:**
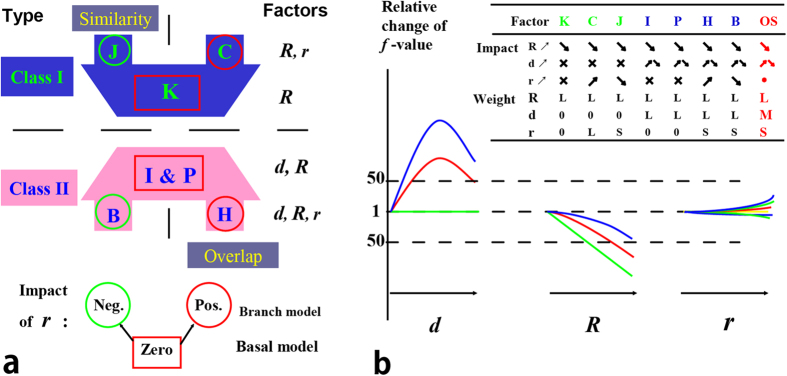
Classification of models by their different weights on parameters. (**a**) Models were separated into two classes by containing d or not. Each class has three kinds of models: the basal model that assigns zero-weight on the minor factor *r* stays in the middle, the left two branch models at either end. (**b**) The impact of every parameter to the function value and their different weights (L: large, M: medium and S: small).

We defined three characteristic parameters (*d, R, r*) to determine three different features of an overlap (see Method). Every (*d, m, n*) triple could be transformed to a (*d, R, r*) triple. As shown in Table 1, the differences among the 6 overlaps in Fig. 1(a) are much clearer shown by the (*d, R, r*) triple than by (*d, m, n*) triple. In addition, the (*d, R, r*) coordinate system gives us an opportunity to classify the models, since five of the seven functions have less variables (see Equations (1~8) in Method). There is an apparent distinction between the expressions which separate them into two classes: expressions of models in class II ( models I, P, H, B) contain *d* but those in class I (models K, C, J) do not (Fig. 2(a)).

We found that the three parameters of an overlap had different impacts to its score, which is the function value (*f*-value) of a selected model. Moreover different models assign different weights to the same parameter (Fig. 2(b)). Parameter *R* is the only one variable present in the expressions of all models, and increase of *R* will lead to large reduction of the *f*-value and all models assign large weight to it; parameter *d* is only present in the expressions of models in class II and has large weight. (More details in Supplementary Section 1). Parameter *r* has a complicated impact and its increase leads to three kinds of changes of the *f*-value, namely positive, negative and zero (Fig. 2). Except for model C, all models assign small or even zero weight on *r*, thus we call *r* as a minor factor for the score of overlap. Indeed, model C exaggerates the impact of *r* to a great extent, which could be shown by an extreme case (Set A has 10000 elements and set B has 1 element, and they share 1 overlapping element. Its score calculated by model C will be the highest, i.e. 1). Then as Fig. 2a shown we knew that the three parameters have different functions: *R* is a primary factor for all models and the common feature for all model; *d* is a primary factor for class II models and separates the models into two class; *r* is a minor factor and leads to the difference among the models within a class.

Difference among models within a class is small. Each class has three types of models, the basal model assigning zero weight on *r*, and two branch models assigning negative or positive weight on *r*. Because of the minor effect of *r*, the basal model is a good approximation of both of the two branch models, such as that Poisson is a good approximation of model H (Hypergeometric distribution) and B (Binominal distribution). In fact *r* essentially describes the difference of the two sets that form the overlap, and the weight on *r* distinguish the two close relations “similarity of two sets” and “overlap of two sets”. Taking cases O3 and O4 in Fig. 1(a) as an example, the two cases have the same *R* and *d*, only the *r* is different. Case O3 consists of two sets of equal size and its *r* is 1, while case O4 consists of two of different size and its *r* is 6.25. Using model J the score of case O3 is larger than that of O4, but the opposite if using model C, and the scores of them are equal if using model K. Then the three kinds of models in a class will be used in different applications. In case that the similarity of two sets is focused, model J should be used; but model C focuses on the overlap of two sets; and both of them could be estimated by model K. Because model C exaggerates the weight on *r* too much and does not take *d* into account, thus we took model H as the most suitable one for measuring overlap.

**Figure 3:**
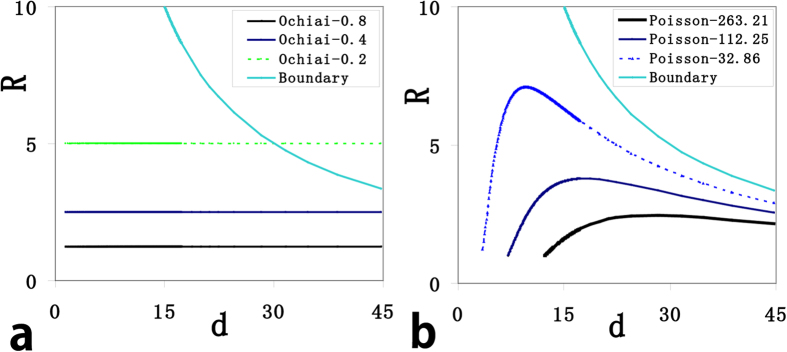
Isolines of different models in the *d-R* plane. (**a**) The isolines of Ochiai, which is a representative and basal model for models in class I. Its isolines are parallel to the *d*-axis since it assigns zero-weight on *d* (does not take *d* into account). (**b**) The isolines of Poisson, which is a representative and basal model for models in class II. Its isolines are curves since it takes both *d* and *R* into account. All its isolines are within the boundary line, case in which has the same *d* and *λ*.

Next we focused on the difference between the two classes of models. We first compared the two basal models, model K (Ochiai) and P (Poisson), and the comparison was done in an *R-d* plane for they have no more than 2 variables. For both models, the isoline near the horizontal axis has the highest score, isolines of Ochiai are horizontal lines (Fig. 3(a)) and isolines of Poisson are curve lines (Fig. 3(b)) within the boundary line. We argued only cases within the boundary line should be focused (see Method). The orientations of isolines tell the weights of parameters, Ochiai assigns zero-weight on *d* (isolines are parallel to the *d* axis) and Poisson assigns large weights on both *d* and *R* (More details in Supplementary Section 1). Other models in class I have similar isolines as Ochiai and those in class II similar as Poisson (see Supplementary Fig. S3~S5), which allow us to take model K (Ochiai) and P (Poisson) as representatives for class I and II respectively in the next analysis.

### Propose OScal to avoid the drawbacks of two classes of models

**Figure 4:**
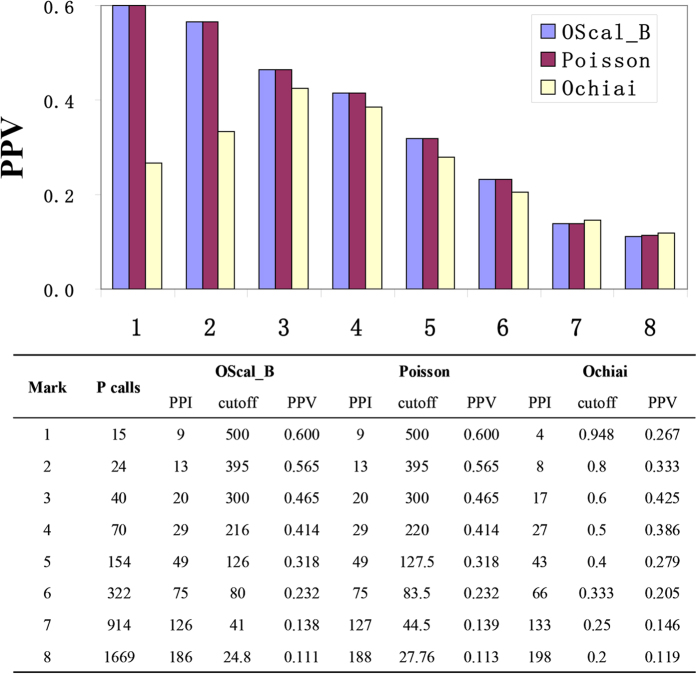
Correlation between PPV and cutoff for different models. The three bars in each group indicate PPVs for the three models when they use different cutoff but select the same amount of positive calls (P calls). The embedded table listed the detailed information.

The two classes of models assign different weights on *d* and *R*, which leads to different performance in application. We took TF-TF data (see Method) to analysis and show the difference between their performances. Every pair of TFs that share at least one common target is modeled by an overlap which is described by an (*d, R, r*) triple. The triples are called as cases and their scores are calculated by the two basal models, namely Ochiai and Poisson. We compared the performance of these two models by the correlation between the score and PPV. Better correlation means better performance, since generally higher-score pairs have higher probability to be functional related TF pairs (true positive). We took PPI pair as an indicator to the true positive and the proportion of PPI pairs in positive calls as an approximation of the positive predictive value (PPV). As shown in Fig. 4, the PPV is not monotone increasing as the increasing score calculated by Ochiai, but there is a well correlation between PPV and the score calculated by Poisson. In short Poisson outperforms Ochiai in sorting the positive calls. We know Ochiai assigns a zero weight on parameter *d*, so it loses the information of *d*. This implies that using both *R* and *d* will generate finer score than using only *R*.

**Figure 5:**
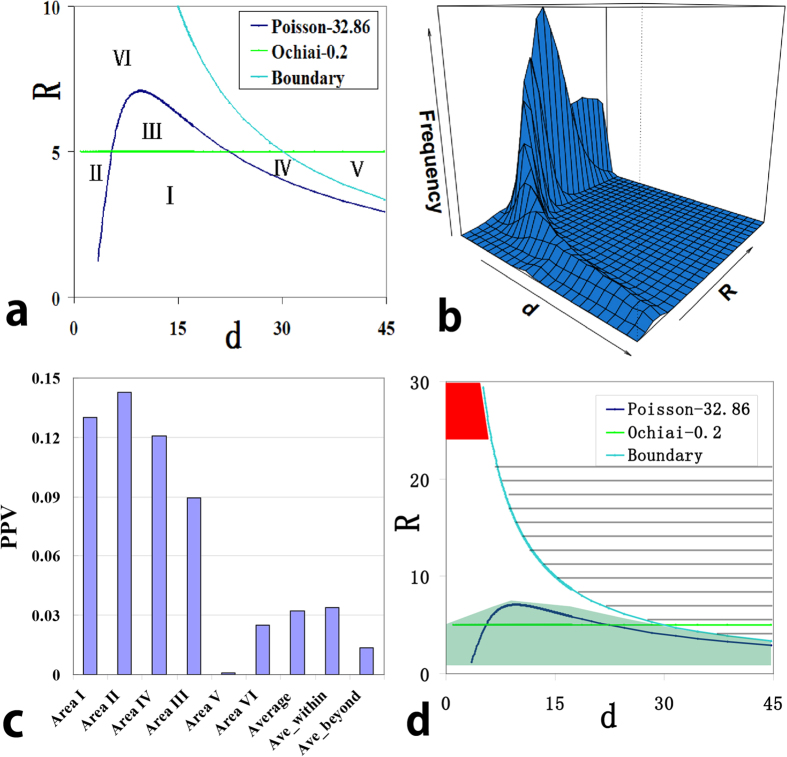
Character of different districts in the *d-R* plane. (**a**) The positive area of Ochiai (district I, II, IV and V) and Poisson (district I and III), cases below the cutoff-line (green for Ochiai, and blue for Poisson) have larger score than the cutoff. (**b**) The uneven distribution of cases in the *d-R* plane. (**c**) The PPV of different districts (Ave_within and Ave_beyond: are the average PPV for the district within and beyond the boundary line respectively). (**d**) The three meaningful areas in the *d-R* plane, i.e. the district beyond the boundary, the low-quality district (red shadow) and the high-quality district (light blue shadow).

Then we further investigated the association of performance between the parameters *d* and *R*. We mapped all the cases of TF data into the *d-R* plane, and found that both the distribution of cases (Fig. 5(b)) and the PPV (Fig. 5(c)) in the *d-R* plane are uneven. Based on these discoveries, three meaningful areas were highlighted as shown in Fig. 5(d). The first one is the district beyond the boundary. We argued cases beyond the boundary should be dropped out and coincidentally all the cases seem to be restricted within it. The second is the low-quality district (LQD, red shadow in Fig. 5(d)), which is characterized by the high-density distribution of low-score cases and low PPV (2.1%, smaller than the average PPV). The LQD should be kept away from the positive area of a good model, since including little part of this district will bring about many false positives. The last is the high-quality district (HQD, light blue shadow in Fig. 5(d)), characterized by the low-density distribution of high-score cases and high PPV (Fig. 5(c)). The HQD mainly consist of the district I~IV and should be covered by the positive area of a good model. More details in Supplementary Section 2.3.

It is interesting that the HQD is very close to the positive area of Ochiai excluding the SR-BB district, implying that *R* is a primary factor and small *R* cases have high probability to be true positives. This is consistent to that *R* is the only one variable present in the expressions of all models. Indeed, Ochiai outperformed Poisson in setting the cutoff which separates the HQD and LQD, which is called as selecting positive calls. It is reasonable to argue that the positive area of a good model should cover the HQD but avoid the LQD as much as possible in the *d-R* plane. Using the cutoff-line shown in Fig. 5(a) (0.2 and 32.86 respectively), Ochiai and Poisson show comparable high PPV (about 12%) but their positive areas are different parts of the HQD. Using lower cutoff (0.141 (*R* = 7.1) and 2.92 respectively, Table 2) to cover the whole HQD, the average PPV of Ochiai and Poisson reduced. The average PPV of Ochiai reduces to 8.5%, but that of Poisson reduced much more greatly to 5.2%. In other words Poisson shows worse performance than Ochiai. We found at this lower cutoff level Poisson will cover part of the LR-Sd district (a LQD) and select too many false positive calls from it, since there is high density of cases as mentioned above. This drawback was called as “LR-Sd trap” in this study. Ochiai also covers part of the district beyond the boundary line, which is another LQD, but the selected false positive calls are very few. So it is not serious in this case, yet we still called this as “SR-BB trap”.

Since the performance mainly dependent on the isolines but regardless of the score for every isoline, so the pros and cons of Ochiai and Poisson showed by the above analysis are also those for other models in each class. In other words, models in class II perform better in sorting positive calls but worse in selecting positive calls, because of the “LR-Sd trap”; models in class I perform better in selecting positive calls (although it will also fall into the “SR-BB trap”) but worse in sorting the positive calls. Here we proposed the OScal model free of such drawbacks, and it consists of three items, a basal model (OScal_B) and two other items (see Method).

**Figure 6:**
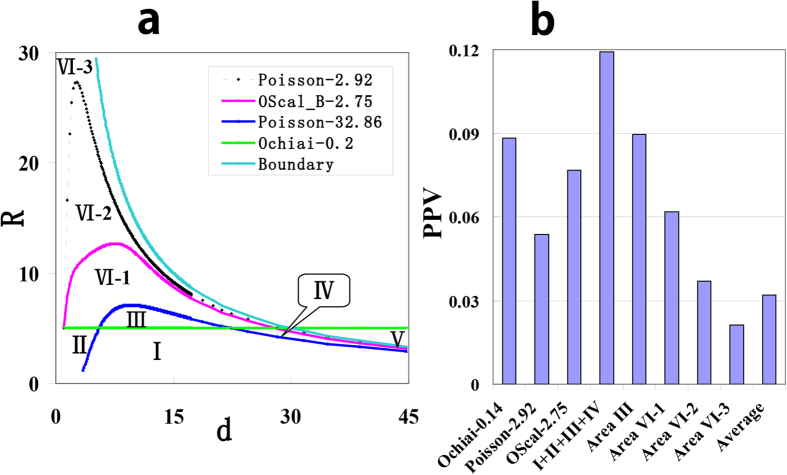
Reasons for different performances. (**a**) The positive area of different models. Using lower cutoff to cover the HQD, the positive area of model P will cover part of LQD, but that of OScal_B will not. (**b**) The average PPV of different models and that of different districts in the *d-R* plane.

The OScal_B was a modification of Poisson, and it is a basal model for OScal. Since Poisson won’t fall into the “SR-BB trap” and have good sorting performance, then only the “LR-Sd trap” is needed to modify. The “LR-Sd trap” is because of the large weight on *d*, since in the isoline (Poisson-2.92 in Fig. 6(a)) there is a sharp increase on *R* when *d* increases from 1 to 25. We found the difference of *d* and *λ* (including *d*/ *λ* and *d*- *λ*, see Method) was the decider for score in Poisson. So we introduced a coefficient to enlarge *λ*. The coefficient is a function of *d* and *R*. It will be large when *d* is small and *R* is large, so the weight on *d* is reduced; it will be near 1 when *d* is large, i.e. *λ* does not change (More details in Supplementary Section 3.2).

As shown in Fig. 4 OScal_B shows comparative performance as Poisson in sorting positive calls. In selecting positive call, using lower cutoff (2.76) to cover the HQD, OScal_B succeeded in avoiding the “LR-Sd trap” and achieve a high PPV (7.5%). At such small cutoff level, OScal_B only selected 6168 cases (Table 2), much fewer than the 14721 cases selected by Poisson. The reason is that OScal_B assigned lower weight on *d* compared to Poisson. The difference between OScal_B and Poisson (Fig. 6(a)) is the district IV-2, which is in the small *d* area. PPV of district IV-2 is as low as the average PPV (Fig. 6(b)), but there are high density cases (8553) in district IV-2 , which is part of the “LR-Sd trap”. Then covering it will introduce many false positive calls, as Poisson does. After taking off the HQD from the positive area of OScal_B at 2.76, district IV-1 is the extra district and its PPV is still relatively high. In other words OScal_B retained the high-quality cases of Poisson but dropped the low-quality cases.

These performances of OScal_B showed it succeeded in giving proper (i.e. medium, Fig. 2(b)) weight on *d*. Heavy weight on *d* lead to the LR-Sd trap as the case in class II (models B, P, H and I) and too small weight on *d* lead to the bad sorting performance as the case in class I (models J, K and C), and OScal_B achieved a good balance between them. In addition we added two items which take minor factor *r* into account. In all OScal takes all the three parameters into account and has similar performance of model H but avoids its drawbacks except for the LR-Sd trap. More details in Supplementary file Section 3.3. When scoring the 6 cases in Fig. 1, OScal is consistent with model H (Table 1).

### OScal shows better performance than existing models in multiple applications

**Figure 7:**
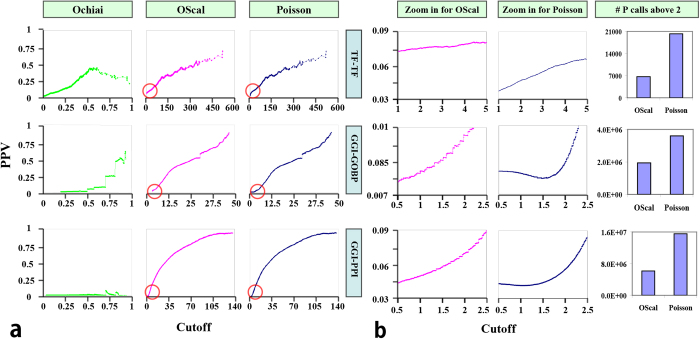
Performances of OScal in different applications. (**a**) Correlation between PPV and cutoff for OScal in three different applications, and those of Ochiai and Poisson are shown for comparing. (**b**) Comparison between OScal and Poisson at low cutoff. The correlation plots are the magnified result in the corresponding area indicating by red circle. The bar chart shows the different number of positive calls for OScal and Poisson taking 2 as cutoff.

OScal was designed to retain the advantages of the two classes of model and avoid their disadvantages, and its basal model (OScal_B) performed better in scoring TF-TF relation. In this section we examined the performance of OScal which takes *r* into account. To avoid the bias of data source, we further added two other applications, i.e. using PPI and GO-BP data to score the gene-gene relation. Two genes sharing many common PPI partners or GO-BP terms would be functional related gene pairs. The score of gene-gene relation were calculated by OScal as well as Ochiai and Poisson, which is used for comparison. The three applications are denoted by “TF-TF”, “GGI-GOBP” and “GGI-PPI” in Fig. 7.

As shown in Fig. 7, OScal always achieve better performance than the other two in all three applications. Figure 7(a) shows it is better than Ochiai and Fig. 7(b) shows better than Poisson. Using OScal there is a good correlation between the PPV and cutoff, which is a major feature of a good model as mentioned above. But the correlation for Ochiai is not very well (bad sorting performance) and further it changes greatly in different applications. Especially, the horizontal correlation curve in GGI-PPI shows that Ochiai generated meaningless scores for the gene correlation using PPI data. Poisson also shows good sorting performance, which is an advantage of it. The plots of OScal are very similar to those of Poisson, showing OScal retained the advantage of Poisson. Indeed OScal is modified from Poisson.

However, there is a clear difference at scores near 0. We zoomed in the area near 0 (indicating by the circle) and the magnified result is shown in Fig. 7(b). The PPV of OScal is larger than that of Poisson at low scores, and the number of positive calls is much smaller than that of Poisson (Bar chart in Fig. 7(b)), implying OScal succeeded in avoiding the “LR-Sd trap” and had few false positive calls. The bar chart shows the number of positive calls for OScal and Poisson at cutoff = 2. The score calculated by Poisson equals 2 means that the occurrence probability of the case is 0.01 by random.

More over OScal avoided another small drawback of Poisson. Poisson can not obtain scores of large overlaps and using it to calculate the scores is very time-consuming. Indeed except for model I all the left three models in class II have such drawback. Because they need to calculate the probability (p-value) first and the p-value of a large overlap calculated by them will be beyond the infimum (see Table 1). This drawback will hinder them to be used widely, especially when scoring the relation of genes using multiple kinds of data, scores of billions of gene pairs need to be calculated. The three p-value-calculating models will fail to obtain scores larger than 300 and it is a heavy load to calculate all the scores using them. For example, when calculating 1 billion cases in a Dell server, Hypergeometric or Binominal distribution needs 10 days, while, OScal just needs 20 minutes. Further the score for any large overlap could be obtained. The expression of OScal is a simple algebraic equation like that of models in class I (e.g. Jaccard index) and it directly calculated the –log (p-value) (see Method). When deal with a few pairs, the calculation could be even manually done in Excel.

## Discussion

Measuring the overlap of two sets is a general and old question^29^,^30^, and many models have been developed. In this study we theoretically compared 7 existing models and proposed our OScal model with better performance in assessing gene relationship.

Our analysis showed there is still space for improvement and new model for measuring overlap is needed. In our study, we selected 7 most representative models to cover a full range of ideas adopted to quantify overlaps. Based on their original purpose they belong to four types: 1) model J and K measure the similarity of two sets; 2) model C measures the overlap; 3) model I measures the mutual information; 4) model P, H and B measure the significance of the overlap. However after redefined by three newly defined (*d, R, r*), they are grouped to only 2 classes by their different weights on *d*, which is an important factor in class II models but not considered by class I models. Difference among models in a class is much smaller than expected and is from the weight on the minor factor *r*. All models take the primary factor *R* into account. Based on such classification, all of the 8 models compared in the study^28^ belong to class I and there is little difference among them. Indeed their performances are very similar based on their result.

The classification distinguished between two types of relationships of two sets: 1) two sets are similar; 2) two sets harbor large overlap. One focuses on the similarity of two sets, and the other focuses on the overlap of two sets. They are two tightly related but different relationships which had been discussed by other studies^31^. Our study identified new contrast between them. We don’t know which relationship of two sets is better for imitating the relationship of real entities. Yet at least this study showed “two sets harbor large overlap” is more suitable for imitating the functional association of genes, and the score using OScal is the best. Of course OScal might be not suitable in the following two cases. The first is that similarity of two entities is concerned very much, and in this case model J in class I would be better. The other is that the overlaps of two sets are very small, and then model H in class II would be better since it is the most sensitive.

In addition, this study showed setting proper weight on parameter *d* is a key task when proposing new model for measuring the overlap. Small weights on *d*, featured by Class I models, will result in bad performance in sorting positive calls and lead the models into the “SR-BB trap”. This told us that *d* contains useful information and should be taken into account. However, models with a heavy weight on *d*, such as Class II models, will fall into the “LR-Sd trap” and fail to properly select positive. Essentially a scoring process contains two related steps: step 1 is selecting positive calls using a proper cutoff and step 2 is sorting the positive calls^32^. Step 1 distinguishes between yes and no, and step 2 distinguishes between good and bad. A good scoring system should have good performances not only at step 1 but also step 2.

Furthermore OScal has not strict restrictive conditions and could be used in a wide range of research areas. The relationship of any pair of entities as long as they share common properties could be measured by OScal. In addition to the applications in this study, OScal could also be used to score the relationship of two miRNAs by their common targets, a TF and a miRNA by their common targets, two gene sets by their overlapping genes, two persons by their common friends and so on. It is noteworthy that gene set enrichment analysis is a very common work in biological study. In essence it is measuring the overlap between two gene sets, one is the query gene list and the other is the GO terms. Fisher exact test or hypergeometric distribution is popularly used to compute the occurrence probability of the overlap assuming it occurs in random. A common problem is that too many GO terms, especially high-level GO terms each of which contains large number of genes, are reported to be enriched terms for a gene list. OScal could be used to measure the overlap and get rid of the drawbacks.

Indeed there are limitations in this study. The first, the ROC curve for OScal is absent for the lack of golden positive and negative standards. Whether for measuring the relation of two sets or two genes, widely-accept high-quality golden standards are in urgent need. Second, OScal is just a practicable model so far, since it is manually constructed with much subjectivity.

In conclusion, this study discovered three characteristic parameters of an overlap and their different weights to its score, and then a more effective model was proposed. This study increased some new knowledge (including new conceptions, new methods and new model) to an old question and deepened our knowledge on overlap or similarity measurement.

## Methods

### Definition of the characteristic parameters (*d, R, r*) for an overlap

Overlap of two sets is determined by the triple data (*d, m, n*). Among them *m* and *n* are the number of elements of the two sets, and *d* is the number of their overlap elements. Except for *d*, the other two are not characteristic parameters of an overlap, but parameters of the original sets. Here we defined three characteristic parameters (*d, R, r*) for an overlap. Their meanings are as the following:

(1) *d*: the size of an overlap, i.e. the number of elements of the intersect;

(2) *R:* average expansion ratio, i.e.


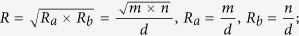


(3) *r*: the difference of the two sets, i.e. *r* = Max(*m, n*)/min(*m, n*).

The three parameters describe three different features of an overlap. A larger overlap not only needs the larger *d* but also the smaller *R*. As shown in Fig. 1, it is clear that the overlap of the two sets in case C is relatively larger than that in case A, although their numbers of overlap are 200.

Then the expressions of the 7 existing functions were re-written with (*d, R, r*), as equation (1~8). Equation 1~8 are expressions of Jaccard index (J), Ochiai coefficient (K), Overlap coefficient (C), Mutual information (I), Poisson (P), Hypergeometric (H) and Binominal (B) distribution respectively. Expressions using (*d, m, n*) are known before, and those using (*d, R, r*) are newly proposed by us.


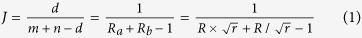



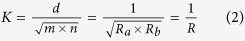



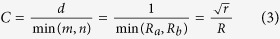



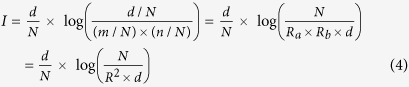



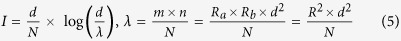



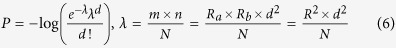



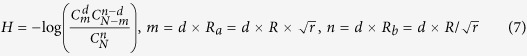



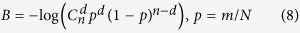


### Construction of OScal

OScal is a modification of Poisson. The overlap score by OScal is the sum of three items: **OS** = **Ps** **+ M**_**J**_**-M**_**r**_. We first use Stirling’s approximation^33^ (eq. (9)) to simplify the expression of Poisson (equation (6)). Stirling’s formula is a powerful approximation for factorials (*d*!), leading to accurate results even for small values of *d*. The approximation of Poisson is as equation (10). The score calculated by Poisson in this study is exactly calculated by equation (10).


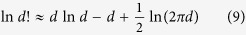



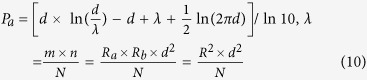


Equation (10) shows that difference between *d* and *λ* (including the ratio and the subtraction) is a decider to the function value. It is known that *λ* is the mathematical expectation of the number of the overlapping elements by random, in other words *d* has the highest probability to be *λ*. When the difference between *d* and *λ* increases, the probability P reduces (P_a_ increases, since P_a_ = –log P). Only the cases *d* > *λ* are thought as “real overlap” in this study. For example, two sets share 1000 overlapping elements, and each of them has 5000 elements, the number of all the background elements (BG number) is *N* = 10000. It is high probability that they have 2500 overlapping by random. But the real number of overlapping is just 1000, so the overlapping elements are too few. In this study, we mainly focus on the cases *d* > *λ,* which are within the boundary line (*d* = *λ*). In the *d-R* plane it is the line *d* = *NR*^−2^.

We found the score in cases with small *d* was overestimated by Poisson. Because when *d* is small, *λ* will be very small, then the difference will be overestimated (appear very large). To modify the overestimation we developed a coefficient to enlarge the *λ* (Supplementary Section 3.2).


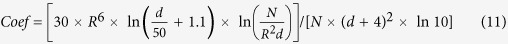


Then the new score P_s_ using enlarged *λ* is calculated by equation (12).


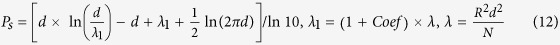


We call P_s_ as OScal_B, which is an approximation of OScal. The expression does not contain *r*. The item M_J_ is used to detect the impact of *r*, and M_r_ is used to detect the hyper-large set (Supplementary Section 3.3).


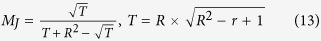



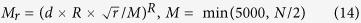


### Simulated data and the three spaces

Since the number of elements is integral, *m*, *n* and *d* must be integer, *d* ≤ *m* and *d * ≤ *n*. Further, any set is a subset of the universal set, so *m* ≤ *N* and *n* ≤ *N*. Here we set N as 22507 (the background number in TF-TF dataset and is the approximate number of genes in human genome). All triple data (*m, n, d*) meet the above criteria were used as simulated data, and their scores were calculated using every function mentioned above. The (*m, n, d*) triple could be changed to (*d, R, r*) triple or (*d, R*_*a*_*, R*_*b*_) triple, each form using corresponding set of coordinates. Then we could know the definition domain for every variable: *R* ≥ 1, 1≤ *r* ≤ *R*^*2*^, *R*_*a*_ ≥ 1, *R*_*b*_ ≥ 1. Each simulated triple data could be mapped to a point in a 3 dimensions space. And the three spaces could be transformed to each other.

### Real data used to compare the performance of models

The data of regulation relation between TF and their targets were downloaded from databases UCSC^34^, TRED^35^ and ChEA^36^. Then we combined them into a whole regulation network containing 300 TFs. The network was used as source data to assess the TF-TF relation. The PPI data were downloaded from HPRD^37^ and BioGRID^38^. The PPI pairs will be used as golden positive standard and also as source data to assess the gene-gene relation at the aspect of PPI (GGI_PPI). The annotated GO Biological Process data of human genes were downloaded from NCBI gene2go to assess the gene-gene relation at the aspect of GO-BP (GGI_GOBP).

### Isoline (or isosurface) and positive area for a model

**Figure 8:**
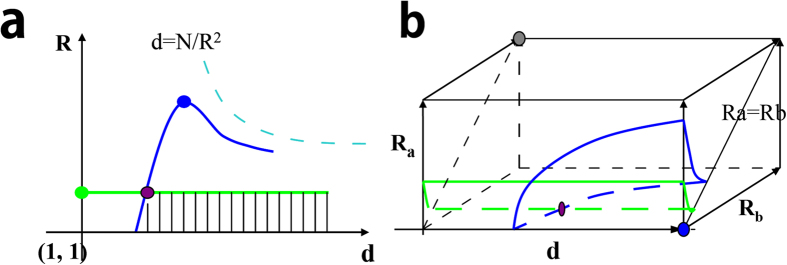
Diagram for isoline or isosurface and the positive area. (**A**) The isoline and positive area for two models in *d-R* plane. (**B**) The isosurface and positive area for two models in the three-dimension space.

Overlap of 2 sets is denoted by a triple data (*d, R, r*). Every triple data is a point in the space and each point has a score using one function. Then we can connect all the points with the same score to form an isoline or an isosurface as shown in Fig. 8. In the application, every model will set a cutoff, so there must be an isoline or an isosurface with the score equal to the cutoff score. Such an isoline or isosurface could be defined as cutoff-line or surface, which divides the space into two parts. One part includes points with higher scores, which is called as positive area. The other part includes all the points with lower scores.

### Determine the impact of parameter by simulation and isolines.

Impact of every parameter to the function value was analyzed using simulation method, and when one parameter is analyzed, the other two were kept constant. Impact has two properties, i.e. direction and intensity. In this study, the direction of impact would be designated as positive, zero or negative, and the intensity of impact designated by qualitative term such as “large, medium, small or zero”. The intensity of impact is also called as “weight”. Isolines could visually show the direction and weight of the impact of every parameter. Large weight on a parameter *x* will lead the isoline to be perpendicular to the *x*-axis (here *x* would be *R*, *d* or *r*). More detailed information see Supplementary Section 1.

### Compare models by positive area in the space.

Models could not be compared directly by the score, since the scores calculated by different models differ greatly, but models could be compared indirectly by their positive area. For example as shown in Fig. 8(a), the blue line is Poisson’s cutoff-line and the green line is Ochiai’s cutoff-line in the *d-R* plane. The area below the blue line is the positive area of Poisson, and that below the green line is the positive area of Ochiai. The difference between Poisson and Ochiai is clearly shown. We call the crosser point of the two cutoff-lines as a reference point, as shown in Fig. 8(a), the purple point is a reference point. Figure 8(b) shows the comparison in three-dimension space, the reference point locates in the cutoff surface. The lower front right corner is the point (the blue point) that has the largest score, the back left corner is the point (the gray point) that has the smallest score.

### Compare models by positive predictive value

Measured by different models, an overlap will get different scores so it is very important to determine which score is appropriate and find out the proper model. Up to now there are no golden standard for similar sets or entities, here we took advantage of the gene-gene relation to compare the scores by different models. There is common idea that functional related genes share similar properties, thus we hypothesize that gene pairs with high similarity scores have high probability to be functional related gene pairs. For the lack of true positive for functional related gene pairs, we take PPI as an indicator to the true positive (TP) based on the hypothesis that PPI pairs have high probability to be functional related gene pairs. If there is high proportion of PPI among the pairs with higher scores calculated by one model, we think this model generates effective scores. Then we take the proportion of PPI as a measure to evaluate the performance of models, and we call it as PPV (positive predictive value) in this study, since the PPV is the ratio of the TP to the positive calls (PC), i.e. PPV = TP/PC, which is very similar to the proportion of PPI.

We compared the models in three different applications, including scoring the TF-TF correlation by their shared targets, the gene-gene correlation by their shared GO biological process terms, and gene-gene correlation by their PPI partners. PPI were thought as the functional related gene pairs in the three applications. If two TFs have many common targets, they would physically interact with each other at a high probability. Two proteins interacting with each other will share many common PPI partners or GO BP terms. Then the cutoff and its corresponding PPV are plotted as the cutoff-PPV correlation curve as shown in Fig. 7.

## Additional Information

**How to cite this article**: Liu, H. *et al*. Scoring the correlation of genes by their shared properties using OScal, an improved overlap quantification model. *Sci. Rep.* **5**, 10583; doi: 10.1038/srep10583 (2015).

## Supplementary Material

Supplementary Information

## Figures and Tables

**Table 1:** Parameters of the 6 overlaps in Fig. 1 and their scores using different models

Case	*m*	*n*	*d*	*R*	*r*	K	C	J	I	P	B	H	OS
O1	25	25	**20**	**1.25**	**1.00**	0.80	0.80	0.67	0.0166	50	54	59	50.16
O2	250	250	**200**	**1.25**	**1.00**	**0.80**	**0.80**	**0.67**	**0.1000**	**287**	**Inf**	**Inf**	**287.39**
O3	250	250	**100**	**2.50**	**1.00**	0.40	0.40	0.25	0.0400	115	124	134	114.36
O4	625	100	**100**	**2.50**	**6.25**	0.40	1.00	0.16	0.0400	115	118	159	114.44
O5	520	520	**200**	**2.60**	**1.00**	0.38	0.38	0.24	0.0577	164	181	199	163.38
O6	520	520	**40**	**13.00**	**1.00**	**0.08**	**0.08**	**0.04**	**0.0023**	**10**	**10**	**11**	**1.66**

Except for the first column, the column names in bold and italic indicate the parameters and the left designate score calculated by corresponding model, for example K designates score calculated by model K (Ochiai coefficient). The score of case O2 calculated by model B or H is “Inf”, because its occurrence probability calculated by model B and H are so small that exceed the infimum (inf) of an ordinary computer and are thought as 0.

**Table 2:** Performances of different models at different cutoffs

Model	High cutoff			Low cutoff			Remark
	Ochiai	Poisson	Oscal_B	Ochiai	Poisson	Oscal_B	Ochiai
Cutoff	0.2	32.86	32.26	0.141	2.92	2.75	0.1
PPV	0.12	0.12	0.12	0.088	0.052	0.075	0.075
#P calls	1669	1458	1462	3414	14721	6168	4826

# P calls: the number of positive calls using the respective cutoff.

## References

[b1] Evans, WE. & Relling, MV. Pharmacogenomics: translating functional genomics into rational therapeutics. Science 286, 487–491 (1999). 10521338 10.1126/science.286.5439.487

[b2] Irizarry, KJL., Merriman, B., Bahamonde, ME., Wong, ML. & Licinio, J. The evolution of signaling complexity suggests a mechanism for reducing the genomic search space in human association studies. Molecular psychiatry 10, 14–26 (2005). 15618953 10.1038/sj.mp.4001576

[b3] Botstein, D. & Risch, N. Discovering genotypes underlying human phenotypes: past successes for mendelian disease, future approaches for complex disease. Nature genetics 33, 228–237 (2003). 12610532 10.1038/ng1090

[b4] McCarthy, MI. *et al.* Genome-wide association studies for complex traits: consensus, uncertainty and challenges. Nature Reviews Genetics 9, 356–369 (2008). 10.1038/nrg2344 18398418

[b5] Chamaillard M. *et al.* Gene–environment interaction modulated by allelic heterogeneity in inflammatory diseases. Proceedings of the National Academy of Sciences 100, 3455–3460 (2003). 10.1073/pnas.0530276100 PMC152314 12626759

[b6] Liu, H. *et al.* CADgene: a comprehensive database for coronary artery disease genes. Nucleic acids research 39, D991–D996 (2011). 21045063 10.1093/nar/gkq1106 PMC3013698

[b7] Wang, F. *et al.* Genome-wide association identifies a susceptibility locus for coronary artery disease in the Chinese Han population. Nature genetics 43, 345–349 (2011). 21378986 10.1038/ng.783

[b8] Wheelock, CE. *et al.* Systems biology approaches and pathway tools for investigating cardiovascular disease. Mol BioSyst 5, 588–602 (2009). 19462016 10.1039/b902356a

[b9] Barabási, A-L., Gulbahce, N. & Loscalzo, J. Network medicine: a network-based approach to human disease. Nature Reviews Genetics 12, 56–68 (2011). 10.1038/nrg2918 PMC3140052 21164525

[b10] Moreau, Y. & Tranchevent, L-C. Computational tools for prioritizing candidate genes: boosting disease gene discovery. Nature Reviews Genetics 13, 523–536 (2012). 10.1038/nrg3253 22751426

[b11] Aerts, S. *et al.* Gene prioritization through genomic data fusion. Nature biotechnology 24, 537–544 (2006). 10.1038/nbt1203 16680138

[b12] Wu, X., Jiang, R., Zhang, MQ. & Li, S. Network-based global inference of human disease genes. Molecular systems biology 4, 189–200 (2008). 18463613 10.1038/msb.2008.27 PMC2424293

[b13] Oti, M. & Brunner, HG. The modular nature of genetic diseases. Clinical genetics 71, 1–11 (2007). 17204041 10.1111/j.1399-0004.2006.00708.x

[b14] Mitra, K., Carvunis, A-R., Ramesh, SK. & Ideker, T. Integrative approaches for finding modular structure in biological networks. Nature Reviews Genetics 14, 719–732 (2013). 10.1038/nrg3552 PMC3940161 24045689

[b15] Goh, K-I. *et al.* The human disease network. Proceedings of the National Academy of Sciences 104, 8685–8690 (2007). 10.1073/pnas.0701361104 PMC1885563 17502601

[b16] Lerman, G. & Shakhnovich, BE. Defining functional distance using manifold embeddings of gene ontology annotations. Proceedings of the National Academy of Sciences 104, 11334–11339 (2007). 10.1073/pnas.0702965104 PMC2040899 17595300

[b17] Yu, G. *et al.* GOSemSim: an R package for measuring semantic similarity among GO terms and gene products. Bioinformatics 26, 976–978 (2010). 20179076 10.1093/bioinformatics/btq064

[b18] Schlicker, A., Domingues, FS., Rahnenführer, J. & Lengauer, T. A new measure for functional similarity of gene products based on Gene Ontology. BMC bioinformatics 7, 302 (2006). 16776819 10.1186/1471-2105-7-302 PMC1559652

[b19] Wu, X., Pang, E., Lin, K. & Pei, Z-M. Improving the measurement of semantic similarity between gene ontology terms and gene products: Insights from an edge-and ic-based hybrid method. PloS one 8, e66745 (2013). 23741529 10.1371/journal.pone.0066745 PMC3669204

[b20] Dannenfelser, R., Clark, NR. & Ma’ayan, A. Genes2FANs: connecting genes through functional association networks. BMC bioinformatics 13, 156 (2012). 22748121 10.1186/1471-2105-13-156 PMC3472228

[b21] Linghu, B., Snitkin, ES., Hu, Z., Xia, Y. & DeLisi, C. Genome-wide prioritization of disease genes and identification of disease-disease associations from an integrated human functional linkage network. Genome Biol 10, R91 (2009). 19728866 10.1186/gb-2009-10-9-r91 PMC2768980

[b22] Radivojac, P. *et al.* An integrated approach to inferring gene–disease associations in humans. Proteins: Structure, Function, and Bioinformatics 72, 1030–1037 (2008). 10.1002/prot.21989 PMC2824611 18300252

[b23] Levandowsky, M. & Winter, D. Distance between sets. Nature 234, 34–35 (1971).

[b24] Abreu, R. & Zoeteweij, P., Van Gemund AJC. An evaluation of similarity coefficients for software fault localization. In: Dependable Computing *, 2006. PRDC'06. 12th Pacific* Rim *International Symposium on* (ed^(eds). IEEE (2006).

[b25] Rivals, I., Personnaz, L., Taing, L. & Potier, M-C. Enrichment or depletion of a GO category within a class of genes: which test? Bioinformatics 23, 401–407 (2007). 17182697 10.1093/bioinformatics/btl633

[b26] Burr IW. Some approximate relations between terms of the hypergeometric, binomial and Poisson distributions. Communications in Statistics-Theory and Methods 1, 297–301 (1973).

[b27] Kraskov, A., Stögbauer, H. & Grassberger, P. Estimating mutual information. Physical review E 69, 066138 (2004). 10.1103/PhysRevE.69.066138 15244698

[b28] Meyer, AdS., Garcia, AAF., Souza, Apd. & Souz CLd. Jr, Comparison of similarity coefficients used for cluster analysis with dominant markers in maize (Zea mays L). Genetics and Molecular Biology 27, 83–91 (2004).

[b29] Kelley, TL. The measurement of overlapping. Journal of Educational Psychology 10, 458 (1919).

[b30] Tilton, JW. The measurement of overlapping. Journal of Educational Psychology 28, 656 (1937).

[b31] Lawlor, LR. Overlap, similarity, and competition coefficients. Ecology 61, 245–251 (1980).

[b32] Hanley, JA. & McNeil, BJ. The meaning and use of the area under a receiver operating characteristic (ROC) curve. Radiology 143, 29–36 (1982). 7063747 10.1148/radiology.143.1.7063747

[b33] Mermin, ND. Stirling’s formula! American Journal of Physics 52, 362–365 (1984).

[b34] Fujita, PA. *et al.* The UCSC genome browser database: update 2011. Nucleic acids research 39, D876–D882 (2011). 20959295 10.1093/nar/gkq963 PMC3242726

[b35] Jiang, C., Xuan, Z., Zhao F. & Zhang, MQ. TRED: a transcriptional regulatory element database, new entries and other development. Nucleic acids research 35, D137–D140 (2007). 17202159 10.1093/nar/gkl1041 PMC1899102

[b36] Lachmann, A. *et al.* ChEA: transcription factor regulation inferred from integrating genome-wide ChIP-X experiments. Bioinformatics 26, 2438–2444 (2010). 20709693 10.1093/bioinformatics/btq466 PMC2944209

[b37] Prasad, TSK. *et al.* Human protein reference database—2009 update. Nucleic acids research 37, D767–D772 (2009). 18988627 10.1093/nar/gkn892 PMC2686490

[b38] Chatr-aryamontri, A. *et al.* The BioGRID interaction database: 2013 update. Nucleic acids research 41, D816–D823 (2013). 23203989 10.1093/nar/gks1158 PMC3531226

