# Supplementary material for: Scoring the correlation of genes by their shared properties using OScal, an improved overlap quantification model
Source: Sci Rep. 2015 May 27;5:10583. doi: 10.1038/srep10583 (PMC4445036; doi:10.1038/srep10583)
Supplement: Supplementary Information [file srep10583-s1.pdf]

Supplementary information

Scoring the correlation of genes by their shared properties using OScal, an improved overlap quantification model

Hui Liu[1 $ *], Wei Liu[1$], Ying Lin[1], Teng Liu[1], Zhaowu Ma[1], Mo Li[1], Hong-Mei Zhang[1], Qing Kenneth Wang[2, 3] and An-Yuan Guo[1*]

1 Department of Biomedical Engineering, Key Laboratory of Molecular Biophysics of the Ministry of Education, College of Life Science and Technology, Huazhong University of Science and Technology, Wuhan, Hubei, 430074, PR China

2 Key Laboratory of Molecular Biophysics of the Ministry of Education, College of Life Science and Technology and Center for Human Genome Research, Huazhong University of Science and Technology, Wuhan, 430074, China

3 Center for Cardiovascular Genetics, Cleveland Clinic, Cleveland, OH, USA

***To whom correspondence should be addressed:**

An-Yuan Guo, Ph.D.

Department of Biomedical Engineering

College of Life Science and Technology

Huazhong University of Science and Technology, Wuhan, HuBei, 430074, China

Phone: 86-27-8779-3177

Fax: 86-27-8779-3177

E-mail: guoay@mail.hust.edu.cn

Correspondence may also be addressed to

Hui Liu, Ph.D.

College of Life Science and Technology

Huazhong University of Science and Technology, Wuhan, HuBei, 430074, China

Phone: 86-27-8779-3177

Fax: 86-27-8779-3177

E-mail: liuhuihust2008@aliyun.com

Guide

There are three sections that deal with different issues in this file:

Section 1. Comparing and analyzing models by their solution space

Section 1.1 Isolines point out the weights and impacts of parameters

Including Fig. S1

Section 1.2 Result of the models in class I

Including Fig. S2~S3

Section 1.3 Result of the models in class II

Including Fig. S4~S7

Section 2. Comparing and analyzing the different performances in application

Section 2.1 Detailed information of the application

Section 2.2 Difference between the positive areas of different models

Including Table S1

Section 2.3 Cases locating in different districts have different quality

Including Fig. S8

Section 2.4 Balance between the two primary factors is important

Section 3. Construction of OScal

Section 3.1 Simplification of Poisson distribution

Including equation (S1~S3) and Table S2

Section 3.2 Construction of the basal model OScal_B

Including equation (S4~S5) and Fig. S9

Section 3.3 Construction of OScal considering the impact of *r*

Including equation (S6~S7), Fig. S10 and Table S3

Section 1. Comparing and analyzing models by their solution space

Overlap of two sets is denoted by a triple data (*d*, *R*, *r*), and evey triple data is a point in the three-dimension space taking (*d*, *R*, *r*) as coordinate system. Generally each point has a score using one function, and the scores should be shown in the fourth dimension. The points having the same score form a surface called isosurface. To show the property using 3-d is much complicated. Fortunately not all functions compared in this study have three variables. If the function has two variables, then the points are mapped into a plane, and their scores are shown in the third dimension, which could be shown by isolines in a plane like a topographic map. Every isoline indicates a score, and the points in the line have same scores. In this study only isolines are studied carefully and the isosurface was just shown by a diagram (Fig. 8 in the main text).

Section 1.1 Isolines point out the weight and impact of parameters

Take the simplest model Ochiai as an example (Fig. S1). Since Ochiai has only one variable, all triples are condensed into a line and their scores are shown in the second dimension (Fig. S1A), showing the score reduces as *R* increase. Its isolines are parallel to the *r* axis in the *R*-*r* plane (Fig. S1B) and *d* axis in the *d*-*R* plane (Fig. S1C), then we would know the weights on *d* and *r* are zero. In the both planes, the isolines are perpendicular to *R* axis and the score indicated in the isoline reduce along this axis. We would know the weight on *R* is large and it has negative impact to the value of *f* (increase of R leads to reduce of *f*).

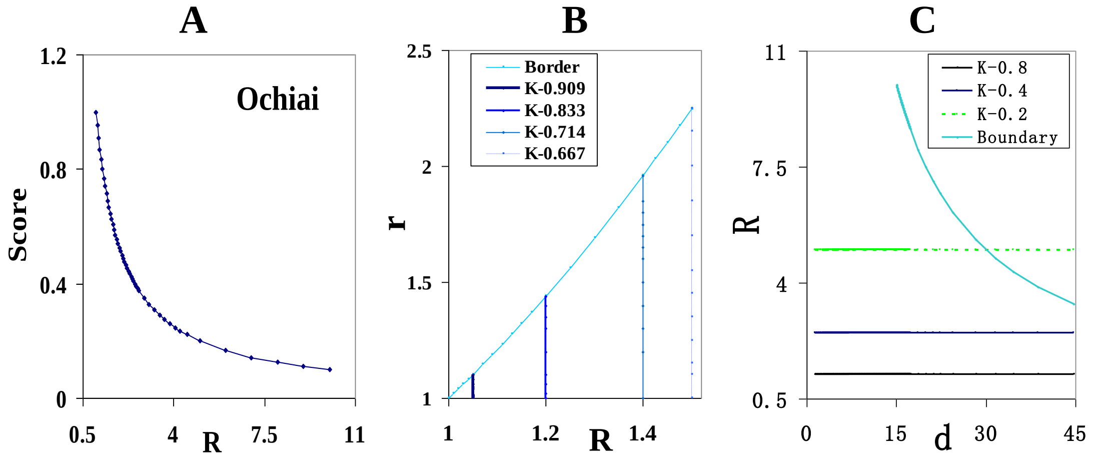

Figure S1 Properties of Ochiai. (A) The score is a function of *R*. (B) Isolines of Ochiai in the *R*-*r* plane. The border line shows the definition space of *r*, which can not be larger than R^2. (C) Isolines of Ochiai in the *d*-*R* plane.

The boundary line is $R^2=N/d$ (which means $d=\lambda$). Because Ochiai does not take *d* into account, its isoline will cross the boundary line.

Although this information could be easily obtained from the expression of Ochiai, which is very simple and only contains *R*, this simple example tells how to get the information from the isolines of a model. Isolines of a model could visually show the weight of every parameter and their impact to the value of *f*.

Section 1.2 Results of models in class I

Models of class I do not contain *d*, and then properties of them could be completely shown by isolines in the *R*-*r* plane. For comparing we put the isolines of the three figures together (Fig. S2).

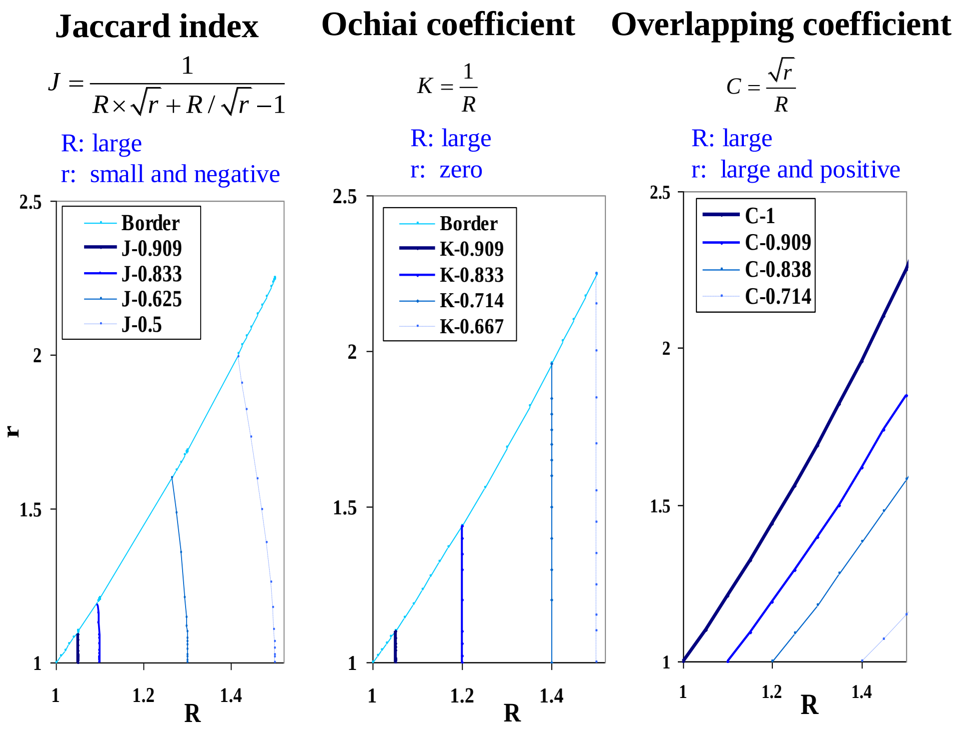

Figure S2 Properties of the three models in class I. Expressions and isolines of every model are shown. And the weights and impacts of *R* and *r* in each model are shown by blue.

The high-score isolines of Jaccard index are nearly parallel to the *r* axis, and the low-score isolines bend a little to the *r* axis, so we know it assign large weight on *R* and small weight on *r*. In addition we would know the impact of *r* is negative, which means increasing of *r* lead to reducing

of the function value. The isolines of Overlapping coefficient are nearly parallel to the diagonal, and then we would know the weights on both of the two parameters are large. Because the isolines bend away from the *r* axis, we know it has positive impact to the function value.

The three modes in class I do not take d into account, so their isolines in *d*-*R* plane are parallel to the *d*-axis. We figure out their isolines in Fig. S3.

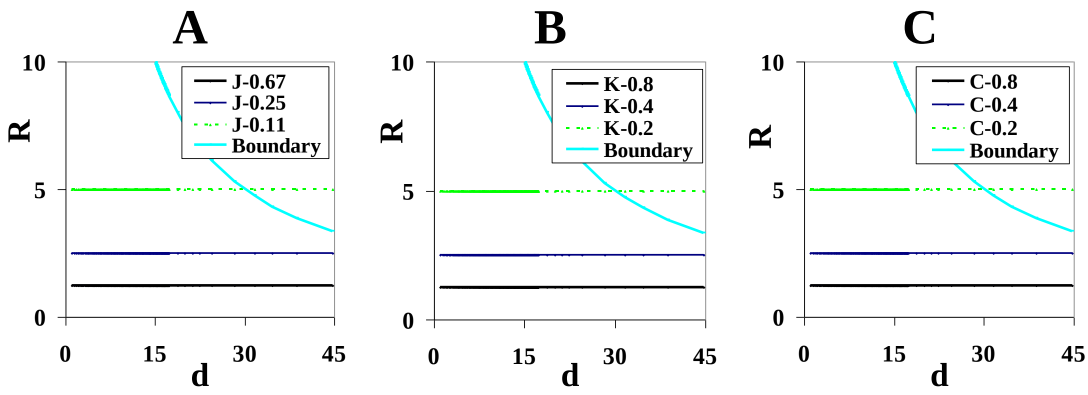

Figure S3 Isolines of models in class I in the *d*-*R* plane. (A), (B) and (C) are result of model J, K and C respectively. They are similar to each other.

Section 1.3 Isolines of models in class II

Comparing different models by their isolines supplies us an opportunity to compare different models which can not be compared before. Mutual information (I) and Poisson distribution (P) are very different models, their scores are heterogeneous. After their isolines are drawn (Fig. S4), it is very clear that they are two similar models, which is also confirmed by their similar expressions. Pa is a simplified form of Poisson distribution using Stirling's approximation. The three isolines of the model I (mutual information) are corresponding to those of model P (Poisson distribution) by three reference points. The two highest-score isolines of them cross at point (150, 1), the two lowest-score isolines cross at point (30, 5), and the left pair of isolines cross at the point (50, 1). It is know that Poisson is an approximation of Binominal and Hypergeometric distributions. As

shown in Fig. S5 the isolines of Binominal and Hypergeometric distributions are very similar to that of Poisson (Fig. S4). Only two of the three reference points mentioned above appear in the Fig. S5. The score of the reference point (150, 1) could not be calculated using these two models. The two highest-score isolines of them cross at point (100, 1).

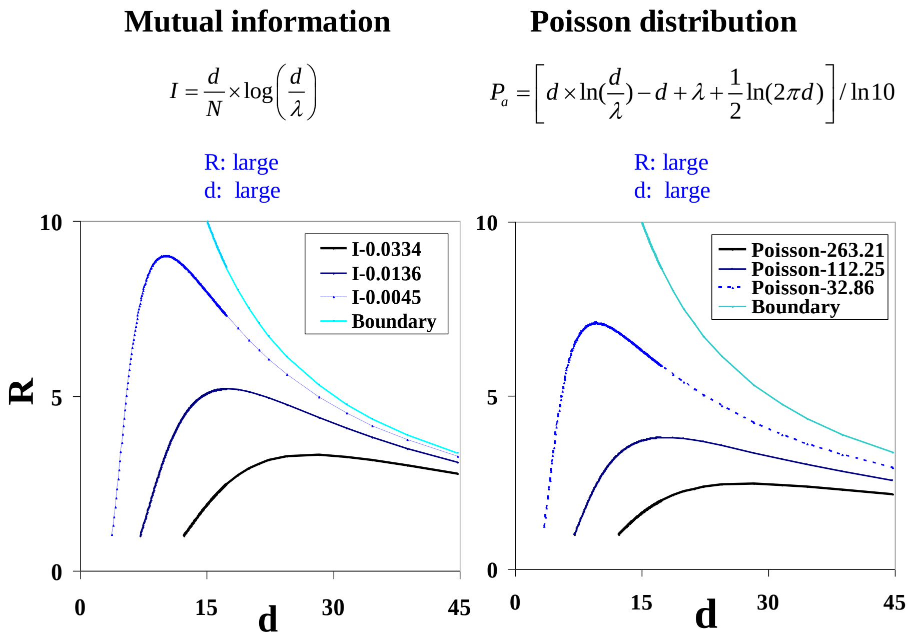

Figure S4 Properties of the two basal models in class II. The isolines of them are within the boundary line. Using model I score of case beyond the boundary will be negative, and in this study only the cases within the boundary are focused.

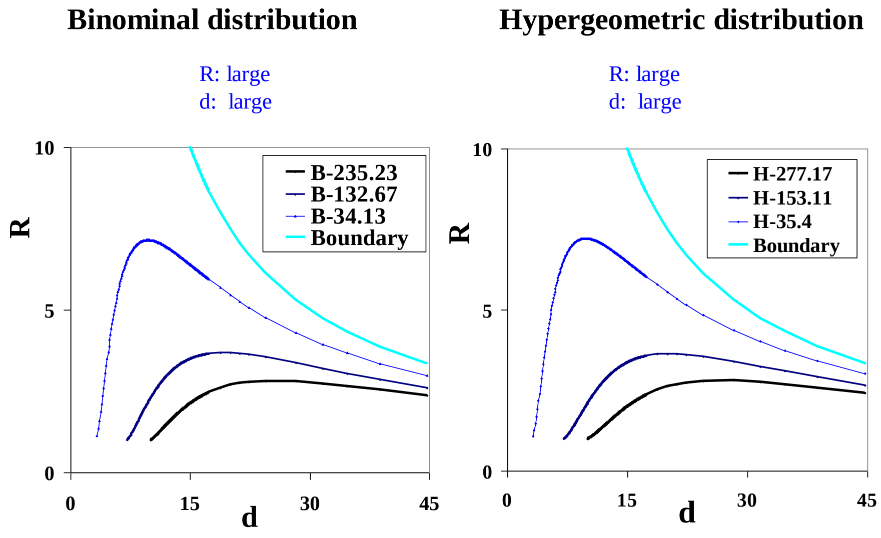

Fig. S5 Isolines of model B (Binominal) and H (Hypergeometric). These isolines are only those in the r=1 plane.

As shown in Fig. S6, among these four models in class II, model I assigns largest weight on *d*, and model P assigns the smallest weight on *d*. Model H and B between them. We put the five isolines of different models together using the reference point (30, 5), i.e. they cross at this point. Model H, B and P are very similar. Since both model B and H take the minor factor *r* into account, and the impact of *r* to the value of *f* was analyzed (Fig. S7).

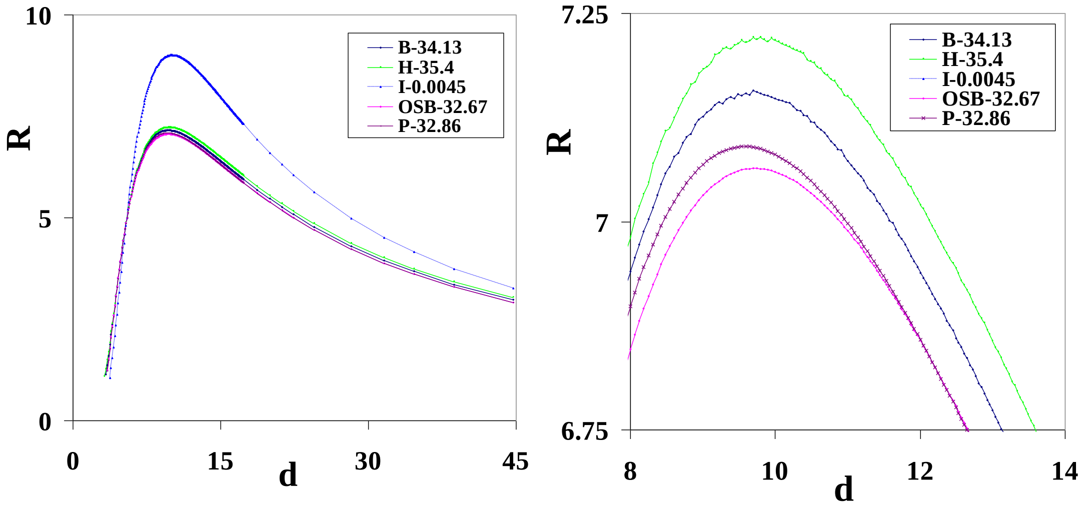

Figure S6 Detailed difference between isolines of models in class II. There are small differences among models in class II.

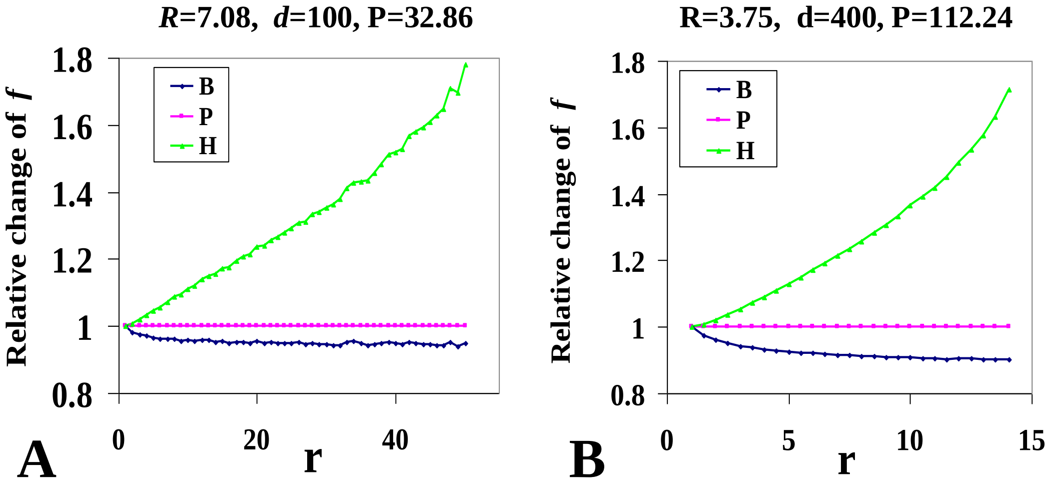

Figure S7 Impact of *r* to the value of *f* for model B, P and H. For model H, the value of *f* increase as *r* increases, but opposite for mode B. Change of *r* lead to small change of *f*.

Section 2 Reasons for different performance of models

Section 2.1 Detailed information of the TF-TF dataset

Transcriptional factors (TFs) are key regulators in biological process, which regulates the temporal and special expression of their targets. Common targets of two TFs were thought to be useful information in assessing the relation of TFs, since if two TFs regulate similar targets they would participate in common biological progresses via regulating similar functional modules. Our TF-TF dataset contain 314 TFs and 22507 targets of them. Any two TFs will be a candidate founctional related pair as long as they share at least one common target, and 41651 candidate pairs are generated. Such criteria are very loose since the randomness degree (the ratio of the number of candidate pairs to all the possible pairs) of this dataset is about 75%. A stricter one is needed to find out true functional related TF pairs. Every TF could be seen as a set of its targets and then every candidate pair could be modeled by two overlapping sets, and the correlation score of the two TFs could be calculated using a model quantifies the overlap. The cases with scores higher than a cutoff will be seen as positive calls. We took PPI pair as indicator to the true positive and the proportion of PPI pairs as an approximation of the positive predictive value (PPV). There are 1334 PPI pairs among all the candidate pairs, and then the average PPV of this dataset is 3.2%.

Section 2.2 Positive area of different models cover different district in the plane

We choose the isoline with the score equal to the cutoff as cutoff-line. Then this isoline of a model could divide the d-R plane into two parts, and the part including points with higher scores is called as positive area of this model. Putting cutoff-lines of different models together, the difference of them will be clearly shown (see Fig. S7A). To describe clearly, we separated the positive areas of

them into different districts by their cutoff-lines: Ⅰ) the small *R* and large *d* (SR-Ld) district; Ⅱ) the small *R* and small *d* (SR-Sd) district; Ⅲ) the larger *R* and large *d* (LR-Ld) district covered only by Poisson; Ⅳ) the small *R* and hyper-large *d* (SR-Hd) district; Ⅴ) the small *R* and beyond the boundary (SR-BB) district. For intuitive feeling, we listed some examples for every district in Supplementary Table S1. Poisson tolerates some larger *R* cases as long as their *d* is large enough, but it will lose some small R cases. Ochiai sticks to *R* strictly since it only takes *R* into account.

Supplementary Table S1. Difference between the positive areas of the two types of models

		**Ⅰ: SR-Ld**	**Ⅱ:SR-Sd**	**Ⅲ: LR-Ld**	**Ⅳ:SR-Hd**	**Ⅴ:SR-BB**
Example	*d-m-n*	**(100, 200, 200)**	**(10, 15, 15)**	**(100, 550, 550)**	**(900, 4050, 4050)**	**(2025, 8100, 8100)**
	d-R-r	**(100, 2, 1)**	**(10, 1.5, 1)**	**(100, 5.5, 1)**	**(900, 4.5, 1)**	**(2025, 4, 1)**
Model	Class Ⅰ	√	√		√	√
	Class Ⅱ	√		√		

Section 2.3 Cases located in different districts have different quality

We mapped all the candidate TF pairs into the *d-R* plane and counted their distribution (Fig. S8B). It is clear that distribution of cases in the high-score area is few, and there is high density of distribution of case in the large R and small d district. Taken the distribution of PPI pairs (taken as true positives) together, we found PPV of different districts is significantly different. Three special districts are highlighted. As shown in Fig. S7C, the PPV of the district beyond the boundary line is very low (1.3%) comparing to the average PPV for all candidate pairs (3.2%). We argued that cases beyond the boundary should be dropped out and all the cases seem to be restricted within it coincidentally. Very few candidate cases locate in the district beyond the boundary line and even fewer PPI pairs locate in this district. This is the first one and the second is the LR-Sd district (the peaks in Fig. S8B). There is high density distribution of cases, and the scores of the cases in this

district are low. We fount the PPV of this area is very low (2.1%). These three characters (high-density, low score, low PPV) imply that this district is a low-quality district (LQD, red shadow in Fig. S8D). Of course there must be a high-quality district (HQD). We have known that near the *d* axis is a high-score district, which will be a little different using different model (Fig. S8A), and there is low density of cases (Fig. S8B). The PPVs of districts Ⅰ-Ⅳ are high (Fig. S8C). Although the two models cover different districts, both of them achieve high PPV (12%) using the cutoff shown in Fig. S8A. We combined their high-score districts and took the union as HQD (light blue shadow in Fig. S8D)

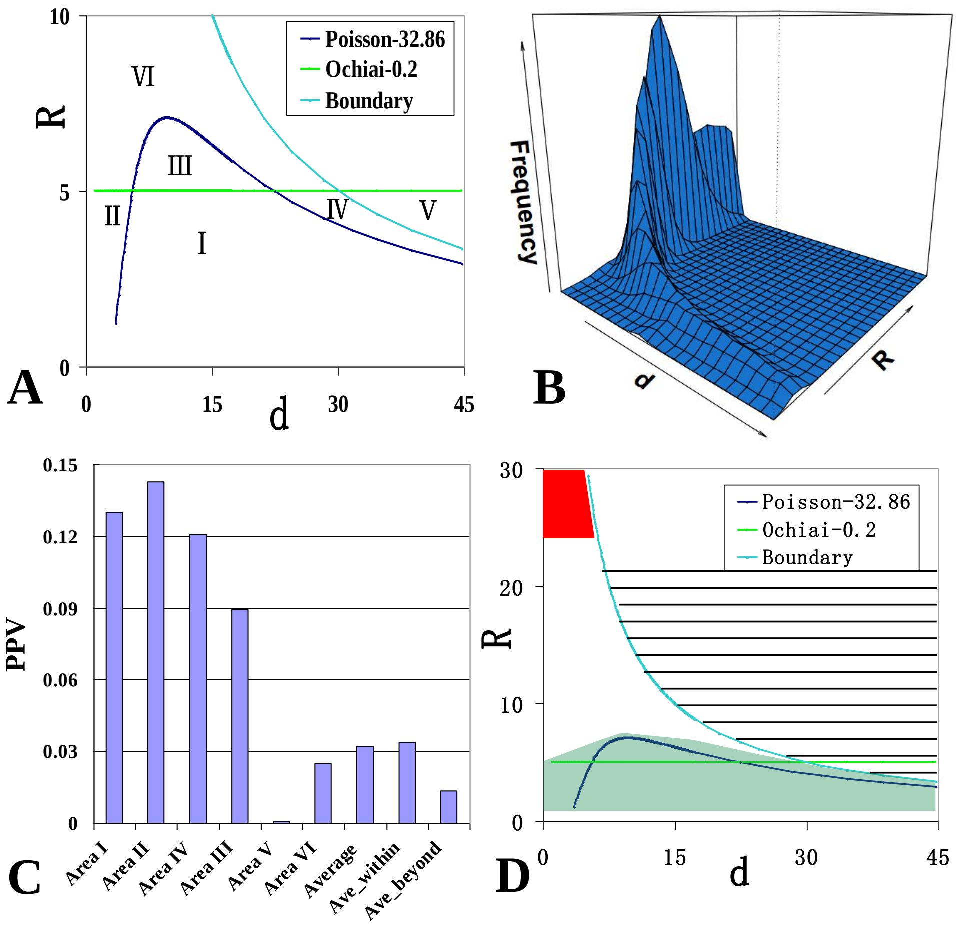

Figure S8 Properties of different area in the *d*-*R* plane using TF-TF data. This is also Fig. 6 in the main text.

Section 2.4 Reasonable balance between d and R is important

The two classes of models assign different weights on *d* and *R*, and then their positive area cover

different districts in the *d*-*R* plane, which leads to different performance in application. Which one is better? We know that an effective model should have two features. First there should be a good correlation between the score and accuracy rate (**Sorting the positive calls**). As mentioned in the main text Poisson is better than Ochiai in this case. Second it should achieve a reasonable balance between sensitivity and specificity using a proper cutoff (**Selecting more true positives but less false positives**). An ROC curve is needed to find out such proper cutoff for a model, and it could also effectively tell the best one among models. However, lack of golden positive and negative standards hindered us to plot the ROC curve. We transformed the second feature into a graphical notion, i.e. the positive area of a good model should cover the HQD but avoid the LQD as much as possible in the *d*-*R* plane. In the main text, we have found that Ochiai is better than Poisson on the second feature.

As shown in Fig. S8D, the HQD is nearly the positive area of Ochiai, which only take *R* into account and does not care about *d*. This implies that parameter *R* is a primary factor and small-*R* is a necessary condition for large overlap (**selecting positive cases**). But small-*R* is not a sufficient condition for large overlap, since we have known that there is bad correlation between PPV and score calculated by Ochiai, which lose some useful information contained in *d*. Poisson takes both *R* and *d* into account and shows better performance in sorting positive calls. But Poisson emphasized *d* too much. A model that achieves reasonable balance between *R* and *d* will lead to better performance.

Section 3 Construction of OScal

OScal is a modification of Poisson and imitates the performance of Hypergeometric distribution. First, we constructed a basal model OScal_B that use only primary factors (*R* and *d*) for OScal. OScal_B is designed to achieve reasonable balance between *R* and *d*, which determine two direct features of an overlap and are two primary factors for measuring overlap. Like Poisson is an approximation of Hypergeometric distribution, OScal_B is also an approximation of OScal. Then we added items that concern the impact of the minor factor (*r*). The expression of OScal contains three items: $\mathbf{OS = Ps + M_J - M_r}$.

Section 3.1 Simplification of Poisson distribution

We used Stirling's approximation (equation (S1)) to simplify the expression of Poisson (equation (S2)) and obtained the approximation expression of Poisson (equation (S2)). The simplified form calculates the scores more quickly and widely than the original express of Poisson distribution. There is very little error between scores calculated by them for a case (Supplementary Table S2), and the simplified form could calculate the score of large overlap (like case SO1) whose score can not be calculated by Poisson. Indeed the scores using Poisson distribution are exactly calculated using this simplified form. P_a is –log (P).

$$\ln d! = d \ln d - d + \frac{1}{2}\ln\left(2\pi d\right) \qquad \text{(S1)}$$

$$P = -\log\left(\frac{e^{-\lambda}\lambda^{d}}{d!}\right), \lambda = \frac{m \times n}{N} = \frac{R_a \times R_b \times d^2}{N} = \frac{R^2 \times d^2}{N} \qquad \text{(S2)}$$

$$P_a = \left[d \times \ln(\frac{d}{\lambda}) - d + \lambda + \frac{1}{2}\ln(2\pi d)\right] / \ln 10, \lambda = \frac{m \times n}{N} = \frac{R_a \times R_b \times d^2}{N} = \frac{R^2 \times d^2}{N} \qquad \text{(S3)}$$

Supplementary Table S2. Difference between scores using model P and P_a for same cases

Case	*m*	*n*	*d*	*R*	*r*	P	Pa
SO1	500	500	500	1	1	Inf	616.10
SO2	200	200	200	1	1	Inf	325.72
SO3	100	100	100	1	1	193.39	193.39
SO4	1000	1000	500	2	1	Inf	329.54
SO5	400	400	200	2	1	207.62	207.62
SO6	200	200	100	2	1	133.77	133.77

Equation S3 shows that difference between d and λ (including the ratio and the subtraction) is a decider to the function value. It is known that λ is the mathematical expectation of the number of the overlapping elements by random, in other word d has the highest probability to be λ. When the difference between d and λ increases, the probability P reduces (P_a increases, since P_a= –log P). If $d>\lambda$, then we thought the overlap is large; if $d<\lambda$, the overlap is very small. For example, two sets share 1000 overlapping elements, and each of them has 5000 elements, the number of all the background elements (BG number) is N=10000. It is high probability that they have 2500 overlapping elements by random. But the real number of overlapping is just 1000, so the overlapping elements are very few. In this study, we mainly focus on the cases $d>\lambda$, which are within the boundary line ($d=\lambda$). In the d-R plane it is the line $d=NR^{-2}$.

Section 3.2 Construction of OScal_B by modification of Poisson distribution

We found the score in cases with small d was overestimated by Poisson. Because when d is small, λ will be very small, then the difference will be overestimated (appear very large). This overestimation will lead to the “LR-Sd trap” of Poisson. We argued that Poisson tolerates too large R, which could be shown by isoline in the d-R plane. As shown in Fig. S9A it is clear that slope of the isoline is very large, especially when d is small.

To modify the overestimation we developed a coefficient to enlarge the λ. Then the new score P_s

for the enlarged λ (λ_1) is calculated using equation (S4). The coefficient should be a function of *d* and *R*, which guarantee Ps is a function of only *d* and *R*.

$$P_s = \left[d \times \ln(\frac{d}{\lambda_1}) - d + \lambda_1 + \frac{1}{2}\ln(2\pi d) \right] / \ln 10, \lambda_1 = (1 + Coef) \times \lambda, \lambda = \frac{R^2 d^2}{N} \quad \textbf{(S4)}$$

There are two guide lines to evaluate the enlarge coefficient. First the coefficient should decrease the slope of the isoline in the *d*-*R* plane, especially when d is small, since we aim to decrease the slope via enlarge *λ* by multiplying a coefficient. Second the new model should still have some tolerance on large *R*. These two guide lines conflict with each other: the more decrease on slop, the less tolerance on *R*. A good one should achieve a balance between them, and at last we find a practicable one. With the coefficient shown in equation (S5), OScal_B succeeded in reducing the slop of the low-score isolines and tolerating some larger *R* (as large as 17).

$$Coef = \left[30 \times R^6 \times \ln(\frac{d}{50} + 1.1) \times \ln(\frac{N}{R^2 d}) \right] / \left[N \times (d+4)^2 \times \ln 10 \right] \quad \text{(S5)}$$

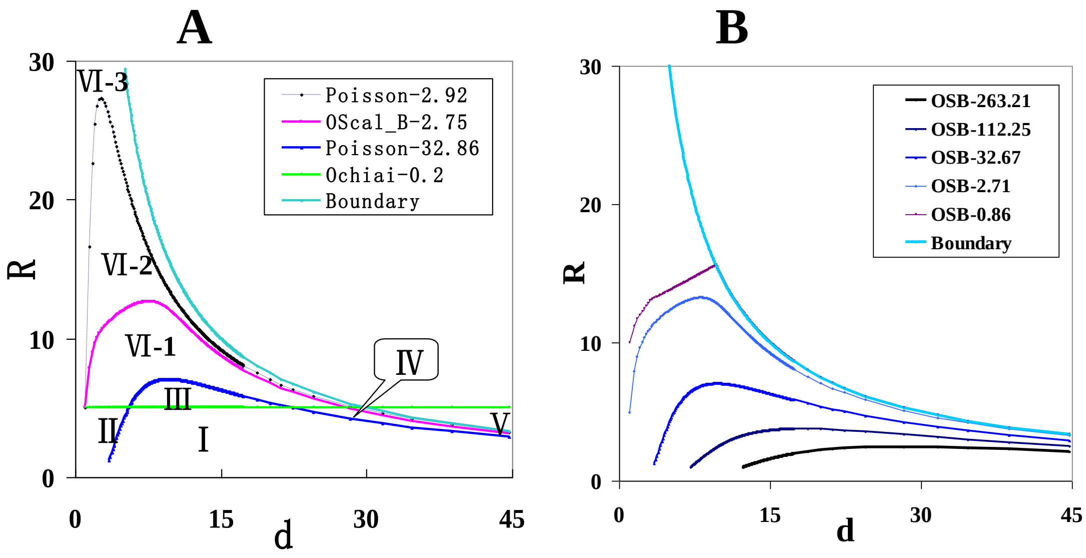

Figure S9 Drawback of Poisson was modified in OScal_B. (A) Positive area of Poisson and OScal_B using lower cutoff to cover the HQD. (B) Isolines of OScal_B in the *d*-*R* plane.

Section 3.3 Construction of OScal taking r into account

Since OScal_B does not take *r* into account, we constructed two other items to contain the impact of *r*. M_J is used to detect the impact of *r*, and M_r is used to detect the hyper-large set.

$$M_J = \frac{\sqrt{T}}{T + R^2 - \sqrt{T}}, T = R \times \sqrt{R^2 - r + 1} \qquad \textbf{(S6)}$$

$$M_r = \left(d \times R \times \sqrt{r} / M\right)^R, M = \min\left(5000, N/2\right) \qquad \textbf{(S7)}$$

In OScal, the impact of *r* will be dependent. When d and R are small, the score will increase as *r* increases as model H does (Fig. S10A). When *d* and *R* are very large, then the score will decrease as *r* increases. Because at this time d*R*sqrt(r) (number of elements in the larger set) will be close to N, in other word the large set is close to the universal set. Table S3 listed some cases generated by such hyper-large sets. The score of case SLO1 using Hypergeometric distribution (H) is large, although the number of the larger set (*m*) is close to the background number (N=22507). Such cases usually appear in enrichment analysis when the high level GO terms are tested. Researchers usually dropped out such high level GO terms when did enrichment analysis using Hypergeomettic distribution. The score of such case using OScal will be negative (Table S3).

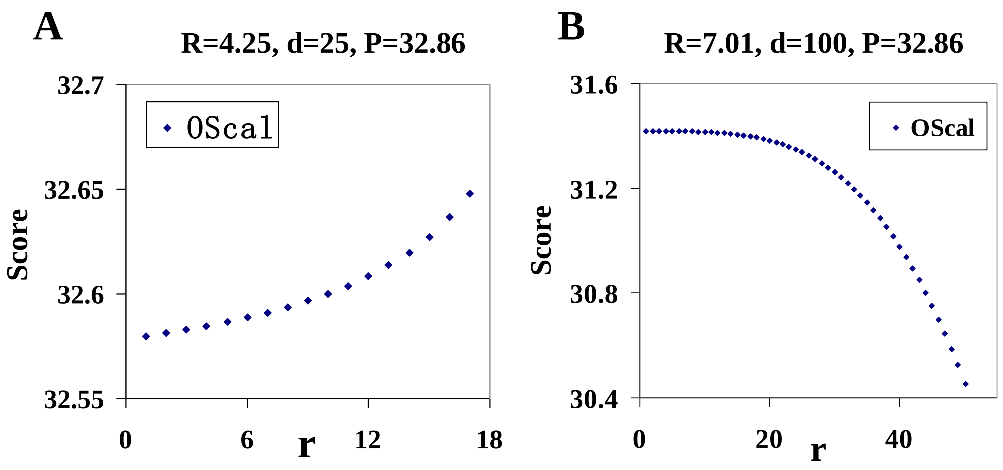

Figure S10 Impact of *r* to score using OScal. Positive (A) and negative (B) impact of *r* to the score in OScal.

Supplementary Table S3. Example of overlaps by hyper-large set and their scores

**Case**	***m***	***n***	***d***	***R***	***r***	**B**	**H**	**P**	**OScal_B**	**OScal**
SLO1	20000	1000	1000	4.47	20	4.94	52.54	4.81	4.81	-489.54
SLO2	18000	900	900	4.47	20	11.35	89.36	10.95	10.95	-298.27
SLO3	16000	800	800	4.47	20	20.71	121.14	19.96	19.96	-163.28
SLO4	14000	700	700	4.47	20	32.37	147.28	31.25	31.25	-70.29
SLO5	12000	600	600	4.47	20	45.48	167.00	44.02	44.02	-7.66
SLO6	10000	500	500	4.47	20	58.96	179.24	57.24	57.24	33.59
SLO7	2000	100	100	4.47	20	67.87	106.13	66.96	66.91	66.15
SLO8	1600	80	80	4.47	20	61.69	92.67	60.93	60.88	60.22
